# A Matrix Metalloproteinase-1 Polymorphism, *MMP1–1607* (1G>2G), Is Associated with Increased Cancer Risk: A Meta-Analysis Including 21,327 Patients

**DOI:** 10.1155/2018/7565834

**Published:** 2018-12-02

**Authors:** Zhonghan Zhou, Xiaocheng Ma, Fangming Wang, Lijiang Sun, Guiming Zhang

**Affiliations:** Department of Urology, The Affiliated Hospital of Qingdao University, Qingdao, China

## Abstract

Although the matrix metalloproteinase-1 (MMP1) polymorphism *MMP1*–1607 (1G>2G) has been associated with susceptibility to various cancers, these findings are controversial. Therefore, we conducted this meta-analysis to explore the association between *MMP1*–1607 (1G>2G) and cancer risk. A systematic search of literature through PubMed, Embase, ISI Web of Knowledge, and Google Scholar yielded 77 articles with 21,327 cancer patients and 23,245 controls. The association between the *MMP1*–1607 (1G>2G) polymorphism and cancer risks was detected in an allele model (2G vs. 1G, overall risk [OR]: 1.174, 95% confidence interval [CI]: 1.107–1.244), a dominant model (2G2G/1G2G vs. 1G1G OR, OR: 1.192, 95% CI: 1.090–1.303), and a recessive model (2G2G vs. 1G2G/1G1G, OR: 1.231, 95% CI: 1.141–1.329). In subgroup analysis, these associations were detected in both Asians and Caucasians. After stratification by cancer types, associations were found in lung, colorectal, nervous system, renal, bladder, and nasopharyngeal cancers. This meta-analysis revealed that *MMP1*–1607 (1G>2G) polymorphism was significantly associated with elevated risk of cancers.

## 1. Introduction

Single-nucleotide polymorphisms (SNP) are variations in single nucleotides that occur at specific positions in the genome and influence protein structure, gene splicing, transcription factor binding, messenger RNA degradation, or sequences of noncoding RNAs [1]. SNPs reportedly contribute to interindividual variability in susceptibility to common diseases such as cancer.

Matrix metalloproteinases (MMPs) are a group of proteolytic enzymes that can degrade extracellular matrix components, thereby affecting various physiological and pathological processes such as embryonic development, wound healing, arthritis, atherosclerosis, and tumor progression [2]. Increasing evidence shows that MMPs play significant roles in cancer development, including cell growth, differentiation, apoptosis, angiogenesis, invasion, and metastasis [3].

MMP1, a member of the MMP family, can degrade interstitial collagen types I, II, and III, clearing a path for cancer cells to invade matrix barriers and migrate through tissue stroma [4]. The *MMP1* gene is located at 11q22.3, and MMP1 expression can be regulated by the *MMP1* promoter. The gene polymorphism *MMP1*–1607 (1G>2G) or rs1799750 in the *MMP1* promoter has been associated with increased susceptibility for various cancers [5, 6]. However, the results were controversial because of variations in cancer types and patient demographics. Therefore, we conducted this meta-analysis to further explore the association between *MMP1*–1607 (1G>2G) polymorphism and cancer susceptibility.

## 2. Materials and Methods

### 2.1. Identification and Eligibility of Studies

We conducted a systematic search of literature published until December 2017 that investigated the association of *MMP1*–1607 (1G>2G) polymorphism with cancer risks, through PubMed, Embase, ISI Web of Knowledge, and Google Scholar, using the terms “Matrix metalloproteinase-1 or MMP-1 or rs1799750,” “polymorphism or variation or mutation or SNP,” and “cancer or carcinoma or tumor or neoplasm.” Only case–control studies with sufficient genotype distribution data to calculate odds ratios (ORs) with 95% confidence interval (CIs) in different gene models were included. Letters, case reports, animal studies, and reviews were excluded. When overlapping populations were included in different articles, only the publication with the largest sample size was selected.

### 2.2. Data Extraction

Two investigators independently reviewed the articles to exclude irrelevant and overlapping studies. The following data were extracted from eligible publications: first author, published year, cancer type, country, ethnicity, control source, genotyping method, and genotype distribution. Any disagreements were resolved by discussion or by consultation with another investigator.

### 2.3. Statistical Analysis

The meta-analysis was conducted using SATAT (version 13.0). The Hardy–Weinberg equilibrium (HWE) for control groups was checked by the chi-square goodness-of-fit test (*P* > 0.05) The associations between *MMP1*–1607 (1G>2G) polymorphism and cancer risks were calculated by OR and 95% CI with the following models to avoid assuming only one suboptimal genetic model: an allele model (2G vs. 1G), a dominant model (2G2G/1G2G vs. 1G1G), and a recessive model (2G2G vs. 1G2G/1G1G). Subgroup analyses were performed by cancer type and ethnicity.

The heterogeneity of studies was assessed by *Q* test using *P* value and *I*^2^ value. A fixed-effects model was adopted when *Q* test indicated a lack of heterogeneity (*P* > 0.05); otherwise, a random-effects model was used. We considered 0–40% of *I*^2^ value to indicate low heterogeneity, 30–60% to indicate moderate heterogeneity, 50–90% to indicate substantial heterogeneity, and 75–100% to indicate considerable heterogeneity. Publication bias was measured with funnel plots and Harbord's and Peter's tests.

## 3. Results

### 3.1. Characteristics of Eligible Studies

**Figure 1:**
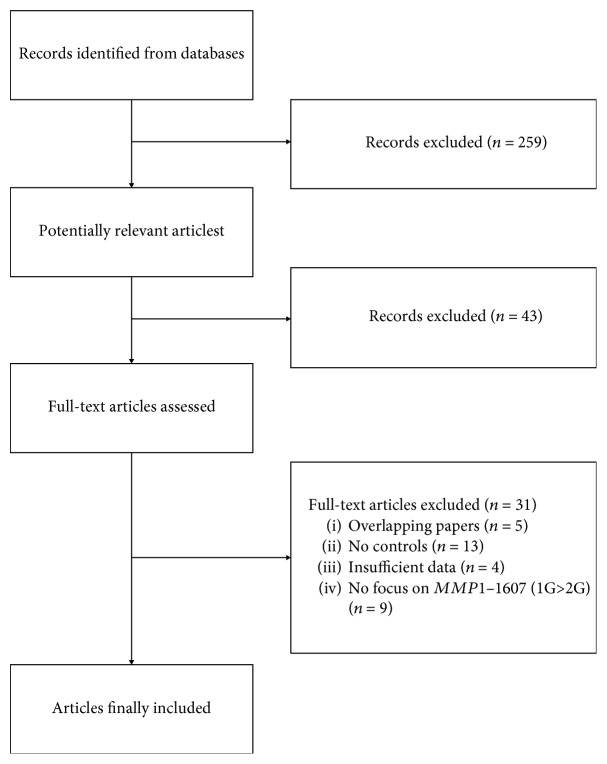
Flow chart of study selection procedure.

The study selection procedure is shown in Figure 1. We included 77 articles with 21,327 cancer patients and 23,245 controls in this meta-analysis (Table 1) [7–83]. Of these, 43 articles were conducted among Asian populations and 34 among Caucasian populations; 67 studies were hospital-based and 10 were population-based. Of the different genotyping methods used in these studies, 45 used polymerase chain reaction-restriction fragment length polymorphism (PCR-RFLP), 18 used TaqMan real-time PCR, 8 used sequencing, and 6 used other methods. Sixteen of the 77 articles showed deviations from HWE in control groups.

### 3.2. Quantitative Analysis

**Figure 2:**
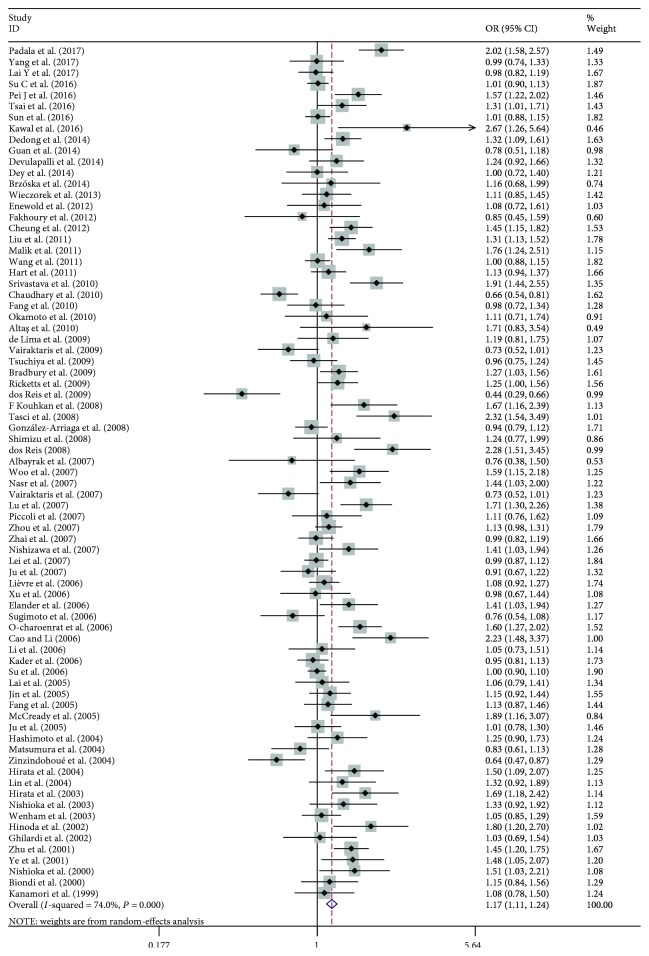
Forest plot of MMP1–1607 (1G>2G) polymorphism and cancer risks in the allele model (2G vs. 1G).

**Figure 3:**
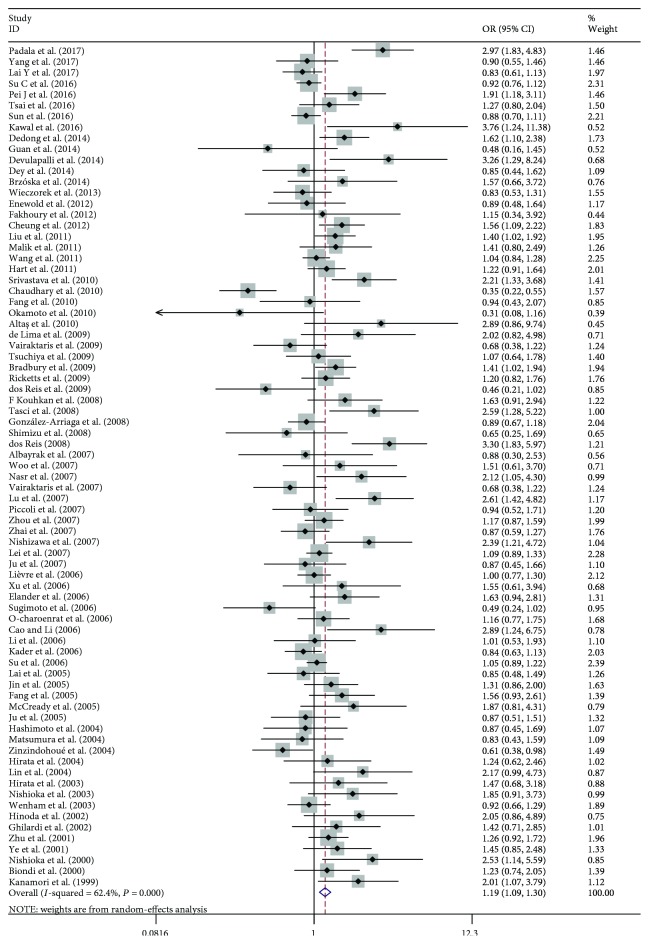
Forest plot of MMP1–1607 (1G>2G) polymorphism and cancer risks in the dominate model (2G2G/1G2G vs. 1G1G).

**Figure 4:**
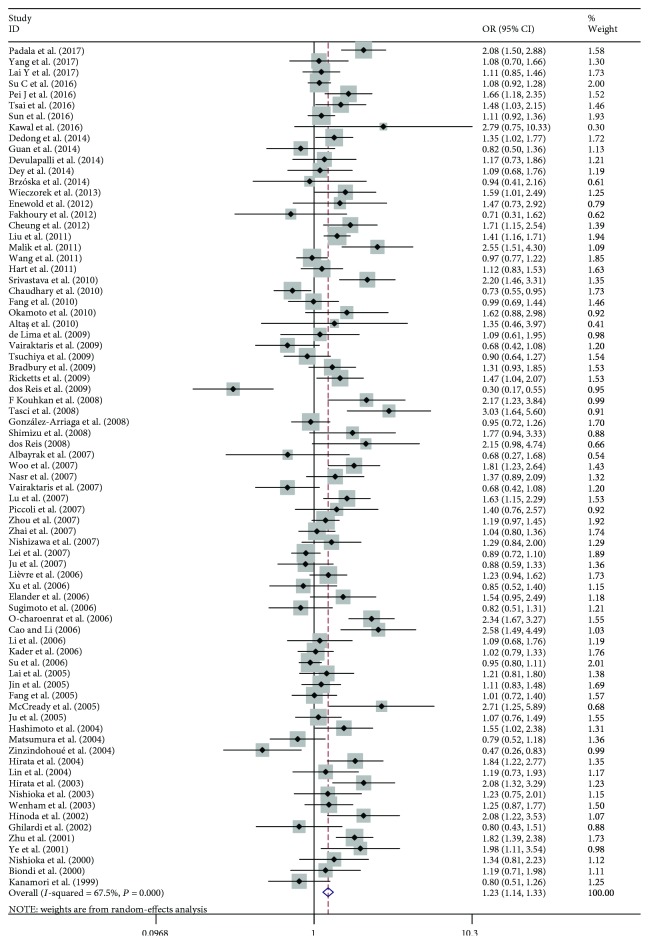
Forest plot of MMP1–1607 (1G>2G) polymorphism and cancer risks in the recessive model (2G2G vs. 1G2G/1G1G).

The main results of this meta-analysis are listed in Table 2. The association between the *MMP1*–1607 (1G>2G) polymorphism and cancer risks was seen in the allele model (2G vs. 1G, OR: 1.174, 95% CI: 1.107–1.244; Figure 2), the dominant model (2G2G/1G2G vs. 1G1G, OR: 1.192, 95% CI: 1.090–1.303; Figure 3), and the recessive model (2G2G vs. 1G2G/1G1G, OR: 1.231, 95% CI: 1.141–1.329; Figure 4).

### 3.3. Risk by Cancer Type

When we considered different cancer types, elevated risk was found in lung cancer in the allele model (2G vs. 1G, OR: 1.128, 95% CI: 1.002–1.268) and the dominant model (2G2G/1G2G vs. 1G1G, OR: 1.127, 95% CI: 1.005–1.264).

Significant association was also found in colorectal cancer in the allele model (2G vs. 1G, OR: 1.279, 95% CI: 1.087–1.505), the dominant model (2G2G/1G2G vs. 1G1G, OR: 1.281, 95% CI: 1.033–1.588), and the recessive model (2G2G vs. 1G2G/1G1G, OR: 1.368, 95% CI: 1.094–1.712).

Five articles addressed the *MMP1*–1607 polymorphism in nervous system cancers, including astrocytoma, glioblastoma, hypophyseal adenoma, and malignant gliomas. Significantly elevated risks were observed in all the three different models (2G vs. 1G, OR: 1.799, 95% CI: 1.493–2.168; 2G2G/1G2G vs. 1G1G, OR: 2.070, 95% CI: 1.474–2.906; and 2G2G vs. 1G2G/1G1G, OR: 1.935, 95% CI: 1.498–2.501).

In renal cancer, the association was found in the allele model (2G vs. 1G: OR: 1.351, 95% CI: 1.149–1.590) and the recessive model (2G2G vs. 1G2G/1G1G OR: 1.674, 95% CI: 1.351–2.073). In bladder cancer, only in the recessive model was significant association detected (2G2G vs. 1G2G/1G1G, OR: 1.739, 95% CI: 1.074–2.816).

Increased risk was also found in nasopharyngeal cancer in the allele model (2G vs. 1G, OR: 1.212, 95% CI: 1.067–1.377) and the recessive model (2G2G vs. 1G2G/1G1G, OR: 1.267, 95% CI: 1.074–1.488).

No relationship was observed in gastric cancer, oral cancer, ovarian cancer, breast cancer, prostate cancer, head and neck cancer, endometrial cancer, hepatocellular cancer, or esophageal cancer (Table 2).

### 3.4. Risk by Ethnicity

In the Asian population, the association between the variation and cancer risks was detected in the allele model (2G vs. 1G, OR: 1.228, 95% CI: 1.130–1.334), the dominant model (2G2G/1G2G vs. 1G1G, OR: 1.256, 95% CI: 1.084–1.456), and the recessive model (2G2G vs. 1G2G/1G1G, OR: 1.297, 95% CI: 1.176–1.431). In the Caucasian population, evaluated risk was also found in the allele model (2G vs. 1G, OR: 1.109, 95% CI: 1.023–1.202), the dominant model (2G2G/1G2G vs. 1G1G, OR: 1.126, 95% CI: 1.015–1.249), and the recessive model (2G2G vs. 1G2G/1G1G, OR: 1.431, 95% CI: 1.013–1.289). Although significant differences were observed in both Asian and Caucasian populations, the Asian population showed higher risk than the Caucasian for the allele, dominant model, or homozygous model, but showed a decreasing trend in the recessive model (Table 2).

### 3.5. Heterogeneity and Sensitivity Analysis

Heterogeneity was observed in overall analyses in all comparison models with *P* < 0.05 and *I*^2^ range from 50.2% to 74.0% (indicating moderate or substantial heterogeneity). We therefore used the random-effects model. Sensitivity analysis to assess influence of individual studies showed no individual study to greatly affect the pooled OR.

### 3.6. Publication Bias

**Figure 5:**
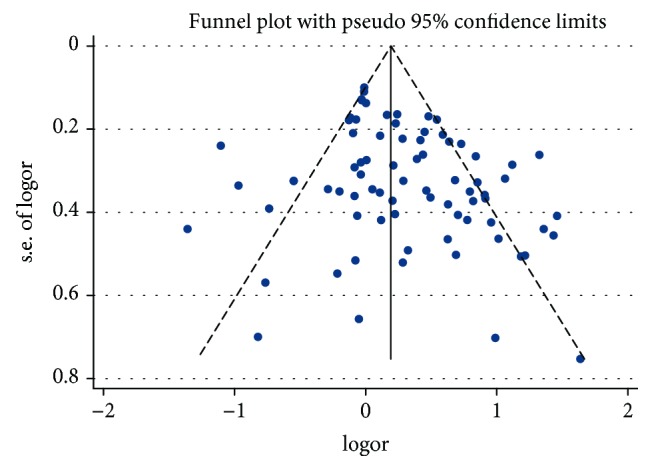
Funnel plot for publication bias test (2G vs. 1G).

The forest plot seemed to be symmetrical (Figure 5). Harbord's and Peter's tests revealed no statistical significance in publication bias (Harbord's: *P* = 0.093; Peter's: *P* = 0.153).

## 4. Discussion

The *MMP1*–1607 (1G>2G) polymorphism has been associated with increased transcription of *MMP1* due to an insert of a guanine base that creates a core-binding site for the EST family of transcription factors, which leads to increased susceptibility for tumor occurrence and progress. The significant association between the variation of *MMP1*–1607 (1G>2G) with some cancer types has been reported by different meta-analyses [3, 4, 84–86].

In the current meta-analysis of 77 articles with 21,327 cancer patients and 23,245 controls, the *MMP1*–1607 (1G>2G) polymorphism was a strong risk factor in various cancers. Although both Asian and Caucasian individuals with 2G alleles or 2G2G genotypes may be more susceptible to cancer development, several studies revealed significant associations in Asians, but not Caucasians [5, 6]. These discrepancies might be due to limited sample sizes. Moreover, the Asian population seemed to show increased risk compared with Caucasian populations when the allele or dominant models were adopted, whereas a decreasing trend was observed in a recessive model, which implies different susceptibilities.

The association was found in lung, colorectal, nervous system, renal, bladder, and nasopharyngeal cancers, but not gastric, oral, ovarian, breast, prostate, head-and-neck, endometrial, hepatocellular, or esophageal cancers, which indicates that the variation plays different roles in various cancers, in accordance with pervious meta-analyses [4, 85, 87, 88]. However, these papers only focused on single types of cancer or one specific ethnicity. Our meta-analysis included all the cancers, analyzed the overall pooled OR, and performed subgroup analyses. Our findings imply a complex relationship between cancer susceptibility and gene variation, influenced by cancer sites and ethnicities.

Recently, the functional studies of SNPs have moved fast. For instance, a study reported that a missense variant rs149418249 in the TPP1 gene confers colorectal cancer risk by interrupting TPP1–TIN2 interaction and influencing telomere length [89]. An expression quantitative trait locus-based analysis revealed that a mutation rs27437, residing in the upstream of SLC22A5, can affect colorectal cancer risk by regulating SLC22A5 expression [90]. Another article reported that a TCF7L2 missense variant rs138649767 associates with colorectal cancer risk by interacting with a GWAS-identified regulatory variant rs698326 in the MYC enhancer [91]. However, the biological mechanisms of functional SNPs still remain challenging. Therefore, further studies are required to promulgate the real functions by which the MMP1–1607 (1G>2G) polymorphism may influence cancer susceptibility and progression.

Our study had some limitations. First, moderate or substantial heterogeneity was detected between studies, which was not significantly decreased by subgroup analysis. When all variations were included in the meta-regression analysis, no obvious factors were detected. More subgroup analyses should be performed, based on factors such as tobacco or alcohol consumption. This conclusion should be interpreted with caution. Second, this analysis was performed with candidate gene strategy in which the MMP1–1607 (1G>2G) polymorphism was selected for study based on a priori knowledge of the gene's biological functional impact on the trait or disease in question [92]. Genome-wide association studies (GWAS) which scan the entire genome for genetic variation include immense amounts of SNPs. Published papers usually reported those SNPs with highly statistical significance (usually *P* < 10^−6^). We have retrieved literature through PubMed in order to search the evidence of association between the MMP1–1607 (1G>2G) polymorphism and cancer risks in GWAS results [92, 93]. However, we did not acquire any positive findings. We speculate that ethnic discrepancy, population stratification, and different standards of statistical significance might lead to negative findings in GWAS. Third, due to the innate shortage of case–control designed studies, the quantity of studies was limited. Third, gene–gene and gene–environment interactions should be considered in analyses of the effects of genes. Fourth, more original papers with large sample sizes were required due to lack of eligible studies in specific cancers in this analysis.

## 5. Conclusions

In conclusion, an association between the *MMP1*–1607 (1G>2G) polymorphism and cancer risks was detected in both Asians and Caucasians. After stratification by cancer types, associations were found for lung cancer, colorectal cancer, nervous system cancer, renal cancer, bladder cancer, and nasopharyngeal cancer. More original studies with larger sample size are required for future analysis.

## Figures and Tables

**Table 1:** The main characteristics of studies included in the meta-analysis.

Author	Year	Cancer type	Country	Ethnicity	Control	Genotype	*N* of case	*N* of control	HWE (*P*)
Kanamori et al. [7]	1999	Ovarian cancer	Japan	Asian	HB	PCR-RFLP	163	150	0.033
Biondi et al. [8]	2000	Other cancer	Italy	Caucasian	HB	TaqMan	160	164	0.813
Nishioka et al. [9]	2000	Endometrial cancer	Japan	Asian	HB	Sequencing	100	150	0.033
Ye et al. [10]	2001	Cutaneous melanoma	England	Caucasian	HB	TaqMan	139	132	0.849
Zhu et al. [11]	2001	Lung cancer	America	Caucasian	HB	PCR-RFLP	456	451	0.028
Ghilardi et al. [12]	2002	Breast cancer	America	Caucasian	HB	Sequencing	86	110	0.652
Hinoda et al. [13]	2002	Colorectal cancer	Japan	Asian	PB	PCR-RFLP	101	127	0.949
Hirata et al. [14]	2003	Renal cell cancer	Japan	Asian	HB	Sequencing	119	210	0.993
Nishioka et al. [15]	2003	Endometrial cancer	Japan	Asian	HB	Sequencing	109	150	0.033
Wenham et al. [16]	2003	Ovarian cancer	America	Caucasian	PB	TaqMan	311	387	0.536
Hashimoto et al. [17]	2004	Head and neck cancer	Japan	Asian	HB	PCR-RFLP	140	223	0.852
Hirata et al. [18]	2004	Renal cell cancer	Japan	Asian	PB	PCR-RFLP	156	230	0.871
Lin et al. [19]	2004	Oral cancer	Taiwan	Asian	HB	Sequencing	121	147	0.336
Matsumura et al. [20]	2004	Gastric cancer	Japan	Asian	HB	PCR-RFLP	215	166	0.432
Zinzindohoué et al. [21]	2004	Head and neck cancer	France	Caucasian	HB	PCR-RFLP	125	249	0.978
Fang et al. [22]	2005	Lung cancer	China	Asian	HB	PCR-RFLP	243	350	0.000
Jin et al. [23]	2005	Gastric cancer	China	Asian	HB	PCR-RFLP	417	350	0.000
Ju et al. [24]	2005	Cervical cancer	Korea	Asian	HB	TaqMan	232	332	0.695
Lai et al. [25]	2005	Cervical cancer	Taiwan	Asian	HB	Other	197	197	1.000
McCready et al. [26]	2005	Glioblastoma	America	Caucasian	HB	PCR-RFLP	81	57	0.916
Cao and Li [27]	2006	Oral cancer	China	Asian	HB	PCR-RFLP	96	120	0.657
Elander et al. [28]	2006	Colorectal cancer	Sweden	Caucasian	HB	Other	127	208	0.918
Kader et al. [29]	2006	Bladder cancer	America	Caucasian	HB	TaqMan	556	555	0.565
Li et al. [30]	2006	Ovarian cancer	China	Asian	HB	PCR-RFLP	122	151	0.008
Lièvre et al. [31]	2006	Colorectal cancer	France	Caucasian	HB	Other	591	561	0.900
O-charoenrat et al. [32]	2006	Head and neck cancer	Thailand	Asian	HB	PCR-RFLP	300	300	0.988
Su et al. [33]	2006	Lung cancer	America	Caucasian	PB	TaqMan	2014	1323	0.597
Sugimoto et al. [34]	2006	Endometrial cancer	Japan	Asian	HB	PCR-RFLP	107	213	0.768
Xu et al. [35]	2006	Colorectal cancer	China	Asian	HB	Other	126	126	0.938
Albayrak et al. [36]	2007	Prostate cancer	Turkey	Caucasian	HB	PCR-RFLP	55	43	0.000
Ju et al. [37]	2007	Ovarian cancer	Korea	Asian	HB	TaqMan	133	332	0.695
Lei et al. [38]	2007	Breast cancer	Sweden	Caucasian	PB	TaqMan	954	947	0.151
Lu et al. [39]	2007	Other cancer	China	Asian	HB	PCR-RFLP	221	366	0.000
Nasr et al. [40]	2007	Nasopharyngeal cancer	Tunisia	Caucasian	HB	PCR-RFLP	174	171	0.091
Nishizawa et al. [41]	2007	Oral cancer	Japan	Asian	HB	TaqMan	170	164	0.493
Piccoli et al. [42]	2007	Renal cell carcinoma	Brazil	Caucasian	PB	PCR-RFLP	99	118	1.000
Vairaktaris et al. [43]	2007	Oral cancer	Greek	Caucasian	HB	PCR-RFLP	156	141	0.276
Woo et al. [44]	2007	Colorectal cancer	Korea	Asian	HB	PCR-RFLP	185	304	0.488
Zhai et al. [45]	2007	Hepatocellular cancer	China	Asian	HB	Sequencing	431	479	0.559
Zhou et al. [46]	2007	Nasopharyngeal cancer	China	Caucasian	PB	Sequencing	829	759	0.634
Dos Reis et al. [47]	2008	Prostate cancer	Brazil	Caucasian	PB	TaqMan	100	100	0.293
González-Arriaga et al. [48]	2008	Lung cancer	Spain	Caucasian	HB	PCR-RFLP	501	510	0.934
Kouhkan et al. [49]	2008	Colorectal cancer	Iran	Asian	HB	PCR-RFLP	150	100	0.935
Shimizu et al. [50]	2008	Tongue cancer	Japan	Asian	HB	TaqMan	69	91	0.585
Tasci et al. [51]	2008	Bladder cancer	Turkey	Caucasian	HB	PCR-RFLP	102	94	0.740
Bradbury et al. [52]	2009	Esophageal cancer	America	Caucasian	HB	TaqMan	313	450	0.508
de Lima et al. [53]	2009	Colorectal cancer	Brazil	Caucasian	HB	PCR-RFLP	108	108	0.258
dos Reis et al. [54]	2009	Prostate cancer	Brazil	Caucasian	HB	TaqMan	100	100	0.293
Ricketts et al. [55]	2009	Renal cell cancer	Polish	Caucasian	HB	TaqMan	323	314	0.847
Srivastava et al. [56]	2010	Bladder cancer	India	Asian	HB	PCR-RFLP	200	200	0.190
Tsuchiya et al. [57]	2009	Prostate cancer	Japan	Asian	PB	Sequencing	283	251	0.285
Vairaktaris et al. [58]	2009	Oral cancer	Greek	Caucasian	HB	PCR-RFLP	156	141	0.276
Altaş et al. [59]	2010	Other cancer	Turkey	Caucasian	HB	PCR-RFLP	30	30	0.195
Chaudhary et al. [60]	2010	Head and neck cancer	India	Asian	HB	PCR-RFLP	422	426	0.240
Fang et al. [61]	2010	Colorectal cancer	China	Asian	HB	PCR-RFLP	237	252	0.683
Okamoto et al. [62]	2010	Hepatocellular cancer	Japan	Asian	HB	PCR-RFLP	91	82	0.009
Hart et al. [63]	2011	Lung cancer	Norway	Caucasian	PB	TaqMan	436	434	0.218
Liu et al. [64]	2011	Lung cancer	China	Asian	HB	PCR-RFLP	825	825	0.924
Malik et al. [65]	2011	Glioblastoma	India	Asian	HB	PCR-RFLP	110	150	0.433
Wang et al. [66]	2011	Cutaneous melanoma	America	Caucasian	HB	TaqMan	864	849	0.940
Cheung et al. [67]	2012	Esophageal cancer	Canada	Caucasian	HB	TaqMan	309	279	0.974
Enewold et al. [68]	2012	Lung cancer	America	Caucasian	HB	Other	71	147	0.743
Fakhoury et al. [69]	2012	Lung cancer	Lebanon	Caucasian	HB	PCR-RFLP	41	51	0.218
Wieczorek et al. [70]	2013	Bladder cancer	Poland	Caucasian	HB	TaqMan	240	199	0.022
Brzóska et al. [71]	2014	Lung cancer	Poland	Caucasian	HB	PCR-RFLP	53	54	0.264
Dedong et al. [72]	2014	Gastric cancer	China	Asian	HB	Other	422	428	0.546
Devulapalli et al. [73]	2014	Gastric cancer	India	Asian	HB	PCR-RFLP	166	202	0.000
Dey et al. [74]	2014	Gastric cancer	India	Caucasian	HB	PCR-RFLP	145	145	0.850
Guan et al. [75]	2014	Esophageal cancer	China	Asian	HB	PCR-RFLP	132	132	0.989
Kawal et al. [76]	2016	Breast cancer	Taiwan	Asian	HB	PCR-RFLP	1232	1232	0.004
Pei et al. [77]	2016	Other cancer	Taiwan	Asian	HB	PCR-RFLP	266	266	0.258
Su et al. [78]	2016	Breast cancer	Taiwan	Asian	HB	PCR-RFLP	1232	1232	0.004
Sun et al. [79]	2016	Oral cancer	Taiwan	Asian	HB	PCR-RFLP	788	956	0.029
Tsai et al. [80]	2016	Nasopharyngeal cancer	Taiwan	Asian	HB	PCR-RFLP	176	352	0.278
Lai et al. [81]	2017	Hepatocellular cancer	Taiwan	Asian	HB	PCR-RFLP	298	889	0.008
Padala et al. [82]	2017	Breast cancer	India	Asian	HB	PCR-RFLP	300	300	0.015
Yang et al. [83]	2017	Gastric cancer	Taiwan	Asian	HB	PCR-RFLP	121	363	0.131

HWE: Hardy–Weinberg equilibrium.

**Table 2:** Stratified analyses of MMP1–1607 (1G>2G) polymorphism on cancer risks by random-effects model.

	*n*	2g vs. 1g					2g2g–1g2g vs. 1g1g					2g2g vs. 1g1g–1g2g				
		OR	UCI	LCI	*P*	*I* ^2^	OR	UCI	LCI	*P*	*I* ^2^	OR	UCI	LCI	*P*	*I* ^2^
Overall	77	1.174	1.107	1.244	0.000	74.0%	1.192	1.090	1.303	0.000	62.4%	1.231	1.141	1.329	0.000	67.5%
Cancer types																
Lung cancer	9	1.128	1.002	1.269	0.006	63.1%	1.127	1.005	1.264	0.365	8.4%	1.153	0.953	1.395	0.002	68.1%
Colorectal cancer	8	1.279	1.087	1.505	0.035	53.6%	1.281	1.033	1.588	0.365	8.5%	1.368	1.094	1.712	0.041	52.1%
Gastric cancer	6	1.106	0.964	1.268	0.165	36.3%	1.221	0.884	1.687	0.061	52.6%	1.121	0.967	1.300	0.448	0.0%
Oral cancer	6	1.121	0.849	1.481	0.000	81.8%	1.254	0.790	1.991	0.001	75.9%	1.108	0.807	1.521	0.003	72.3%
Nervous system cancer	5	1.799	1.493	2.168	0.869	0.0%	2.070	1.474	2.906	0.438	0.0%	1.935	1.498	2.501	0.475	0.0%
Ovarian cancer	4	1.022	0.888	1.176	0.845	0.0%	1.090	0.769	1.545	0.174	39.7%	1.013	0.823	1.247	0.417	0.0%
Breast cancer	4	1.194	0.904	1.576	0.000	89.6%	1.352	0.906	2.017	0.000	84.9%	1.149	0.809	1.632	0.000	84.7%
Renal cancer	4	1.351	1.149	1.590	0.328	12.8%	1.179	0.898	1.547	0.829	0.0%	1.674	1.351	2.073	0.580	0.0%
Bladder cancer	4	1.437	0.960	2.152	0.000	89.2%	1.349	0.771	2.360	0.001	83.1%	1.739	1.074	2.816	0.001	81.7%
Prostate cancer	4	0.932	0.485	1.791	0.000	90.3%	1.136	0.493	2.616	0.001	82.5%	0.780	0.375	1.623	0.001	82.3%
Head and neck cancer	4	0.958	0.595	1.543	0.000	92.6%	0.678	0.388	1.186	0.001	81.1%	1.071	0.539	2.219	0.000	92.4%
Endometrial cancer	3	1.147	0.756	1.741	0.020	74.4%	1.312	0.492	3.497	0.005	81.0%	1.091	0.807	1.476	0.320	12.3%
Nasopharyngeal cancer	3	1.212	1.067	1.377	0.340	7.4%	1.299	0.996	1.696	0.319	12.5%	1.265	1.074	1.488	0.535	0.0%
Hepatocellular cancer	3	0.995	0.875	1.131	0.890	0.0%	0.816	0.631	1.055	0.333	9.0%	1.118	0.932	1.341	0.428	0.0%
Esophageal cancer	3	1.189	0.899	1.572	0.039	69.1%	1.321	0.908	1.922	0.138	49.5%	1.260	0.866	1.835	0.080	60.4%
Other cancers	7	1.172	1.010	1.360	0.043	53.8%	1.128	0.903	1.410	0.167	34.2%	1.278	1.038	1.573	0.073	48.0%
Ethnicity																
Asian	43	1.228	1.130	1.334	0.009	75.2%	1.256	1.084	1.456	0.000	68.9%	1.297	1.176	1.431	0.000	66.4%
Caucasian	34	1.109	1.023	1.202	0.009	71.2%	1.126	1.015	1.249	0.000	50.1%	1.431	1.013	1.289	0.000	67.2%

*n*: number of comparison; *P*: *P* value of *Q* test for heterogeneity test; UCI: upper limit of the 95% confidence interval; LCI: lower limit of the 95% confidence interval.
